# Impacts of stocking density rates on welfare, growth, and hemato-biochemical profile in broiler chickens

**DOI:** 10.5455/javar.2021.h556

**Published:** 2021-11-04

**Authors:** Mishkatul Zabir, Mohammad Alam Miah, Mahabub Alam, Md. Eftakhar Jahan Bhuiyan, Md. Iqramul Haque, Khaled Mahmud Sujan, Afrina Mustari

**Affiliations:** Department of Physiology, Faculty of Veterinary Science, Bangladesh Agricultural University, Mymensingh 2202, Bangladesh

**Keywords:** Broilers, hemato-biochemical profile, stocking density, welfare

## Abstract

**Objective:**

The study investigated the effect of different stocking density (SD) rates on the welfare, growth, and hemato-biochemical parameters in broiler chickens.

**Materials and Methods:**

106 broiler chicks of 10 days old were used and assigned into four groups: A, B, C, and D. The chicks of group A were reared in floor space containing one bird per square foot area (SD1.0). The chicks of groups B, C, and D were reared at 1.5, 2.0, and 2.5 birds per square foot area (SD1.5, SD2.0, and SD2.5). Welfare, body weight, and hemato-biochemical parameters were assessed and monitored by physical observation and laboratory methods.

**Results:**

The birds reared at SD2.0, and SD2.5 rates showed increased panting breathing. Wet feces adhered below the vent. There were a significant number of birds showing dirtiness of body and feathers. Birds reared in SD2.5 were familiar with moist litters and high ammonia smell. Foot-pad dermatitis, scratches, and blister formation were detected in the leg. The study revealed that the higher SD negatively correlated to the welfare behavior indicators. Live body weight was significantly (*p < *0.05) decreased in birds reared at higher SD rates. Birds housed in SD1.0 and SD1.5 are optimum for body weight and improved feed conversion ratio. The hemato-biochemical parameters of birds reared at various SD rates did not differ. The total leucocyte count increased significantly, while total serum proteins decreased gradually as SD rates increased.

**Conclusion:**

This work explores that higher SD negatively affects welfare and growth performance in broiler chickens.

## Introduction

Poultry rearing has evolved from backyard rearing to a commercially organized, scientific, and thriving industry. With commercialization, most chickens raised for meat worldwide are raised in a conventional intensive farming system. Such poultry rearing systems are under the radar for the lack of animal welfare implementation. Consumers are concerned about the artificial nature of conventional intensive farming, which lacks the supply of natural lighting and outdoor access for the birds. Commercial broiler producers often rear broilers in high stocking density (SD) situations to achieve higher profits. This may result in insufficient airflow and higher heat accumulation at birds’ micro-climate levels, leading to higher mortality, and lower growth [1,2]. A welfare-based rearing system protects birds from any discomfort, pain, injury, and helps to express their natural behavior. In broiler production, SD is a very important welfare factor. It specifies the number of broiler chickens or the total weight in kilograms in a house per square meter or foot of usable area [3]. Any deviation from the ideal condition can lead to a reduction in performance [2]. High SD can contribute to poor performance due to several factors. However, from a farmer’s point of view, it might seem beneficial to raise more broilers per unit area. The labor cost, housing, feeding, and equipment per bird decreases as the number of broiler birds per given area increases [1]. Nevertheless, high SD in broilers has been linked to lower feed intake, stunted growth, decreased crouching, walking, preening, lower grade poultry products, stress, listlessness, and an increased risk of health problems [4–7]. SD has an impact on growth and leg-related problems by influencing litter and air quality. The high moisture content of the litter promotes microbial activity, which raises the room temperature and ammonia concentrations in broiler houses.

SD affects the stretching behavior of broilers, causing temporal and spatial variations [8,9]. Broiler chickens in the high SD stretched more during the 7 to 10 week period and panted more during 6 and 9 weeks, implying that more outdoor pasture space may be positively linked with improved broiler wellbeing [10]. Broiler chickens housed in monotonous environments at high SDs have been found to be detrimental to their welfare. On the other hand, broilers residing in complex environmental conditions have shown consistent responses with lower anxiety and better welfare [11]. Broilers of the first growing breed reared at 34 kg/m^2^ had the worst health conditions, including maximum mortality rate, worst walking ability, hock burn, pododermatitis, and poor litter quality [12].

Several studies on the impact of SD on broiler growth performance have been undertaken. However, the majority of these findings were not always conclusive. Some studies showed considerable benefits from reducing SD on the performance of broilers [4,13], while others reported that reducing SD did not affect [14] or even had negative effects on broiler growth performance [2]. The disparities among these studies suggest that more focused research is needed to determine how broiler performance is affected by the different SD rates. Recently, much concern has been expressed about the role of SD regarding the welfare aspect of poultry production [15]. The European Commission [European Union (EU)] reported the basic standards for broiler welfare with the highest SD of 30 kg/m^2^ of broiler chickens throughout the EU. Broilers must be provided with an optimal environment and a comfortable temperature in order to maintain an optimal body temperature and achieve their potential for superior growth [16]. The current research investigated the impacts of various SD rates on the welfare, growth, and hemato-biochemical parameters of broiler chickens.

## Materials and Methods

### Ethical approval

The research study protocols were approved and carried out in accordance with the Animal Welfare and Experimentation Ethics Committee (AWEEC) of Bangladesh Agricultural University, Mymensingh, Bangladesh [AWEEC/BAU/2020-19].

### Statement of the experiments

The experiment was done at the Department of Physiology, Faculty of Veterinary Science, Bangladesh Agricultural University, Mymensingh, from 10 February 2020 to 12 March 2020. In the research, 106 Lohman day-old broiler chicks were purchased from a poultry hatchery in Mymensingh and brooded according to the standard protocol described elsewhere.

### Experimental protocol

**Figure 1.**
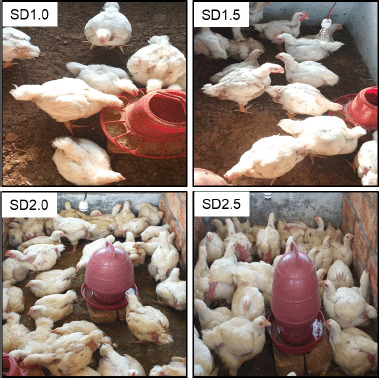
Representative pictures of broilers housed at different SD rates. Broilers raised in higher SD (SD2.0 and SD2.5) had a moist litter and looked dirty.

The experiment was carried out in a completely random manner. On the 10th day, the chicks were separated into four groups: A, B, C, and D, with different SD rates. The broiler chicks of each group were reared separately (Fig. 1).

Group A (SD1.0): 1 bird per square foot area (15 birds in a 15-square-foot area).

Group B (SD1.5): 1.5 birds per square foot (23 birds in a 15-square-foot area).

Group C (SD2.0): 2 birds per square foot (30 birds in a 15-square-foot area).

Group D (SD2.5): 2.5 birds per square foot (38 birds in a 15-square-foot area).

After recording the initial body weight, each bird was kept in separate specified floor space. Furthermore, body weight was recorded weekly until the broiler birds were sacrificed on the 32nd day to collect blood samples for selected hematological studies [total erythrocyte count (TEC), hemoglobin (Hb), and packed cell volume (PCV)] and serum samples for certain biochemical studies [lipid profile, aspartate aminotransferase (AST), alanine aminotransferase (ALT), creatinine].

### Management procedures

Starter, grower pellet, and finisher broiler feeds were purchased from Nourish poultry feed Ltd, Dhaka, Bangladesh. Birds were given a broiler starter for 11 days (0–10 days), grower feed for 13 days (10–22 days), and a finisher diet for the last 10 days (23–32 days). Feed and water were provided on an *ad libitum* basis. The conventional broiler farming technique was closely followed in terms of broiler management and rearing. According to the manufacturer’s recommendations, the birds were immunized against common infectious diseases. During the testing period, strict biosecurity controls were implemented.

### Welfare assessment

The welfare of broiler chickens was evaluated by inspecting litters, moisture conditions, panting behavior, dirtiness, feces adherences, alertness, leg lesions, hematomas, foot-pad dermatitis, scratches and breast blisters in live birds, mortality rate, post-mortem lesions in bird internal organs, and so on [17]. 

### Measurements of live body weight and feed conversion ratio (FCR) 

The live body weight of each bird in each group was measured weekly using the digital balance on the 10th, 17th, 24th, and 32nd days of age, and the total body weight gain was computed by subtracting the initial live body weight from the final live body weight (body weight gain = final body weight – initial body weight). Feed consumption refers to the amount of feed consumed by the birds in a given time period. Feed intake was calculated by subtracting the amount of feed supplied to the birds from the amount of feed remaining at the end of each feeding period. The FCR was determined by dividing the total feed consumed by the total body weight gain (FCR = total feed consumed by the birds/total body weight gain). 

### Blood sampling and serum preparation 

About 10–12 ml of blood was collected from six to eight birds in each group at slaughter after 32 days of the experimental period. Half of the blood was kept in a sterile test tube containing anticoagulant while the remaining half was taken in another sterile test tube containing no anticoagulant for serum preparation [18]. The serum was separated from the clotted blood after 15 min of centrifugation at 1,000 rpm. Serum samples were kept at −20°C for further biochemical analysis.

### Analysis of blood and sera

The standard technique was followed to test the selected blood parameters (Hb, TEC, and PCV) within 1–2 h of the blood collection [18]. Total leukocyte count (TLC) was performed by counting the leucocyte numbers from smear slides stained with modified Wright’s stain under the 40× objective of the compound microscope. The biochemical tests were done at Professor Muhammad Hossain Central Laboratory of Bangladesh Agricultural University, Mymensingh. The total serum cholesterol (TC), triglycerides (TG), high-density lipoprotein-cholesterol (HDL-c), creatinine, ALT, AST, and total protein were performed using a UV spectrophotometer, T 80, by PG Instruments, Great Britain. Specific reagents from High Technology Incorporation in the United States were employed [19].

## Results and Discussion

### Welfare behavior indicators

**Figure 2.**
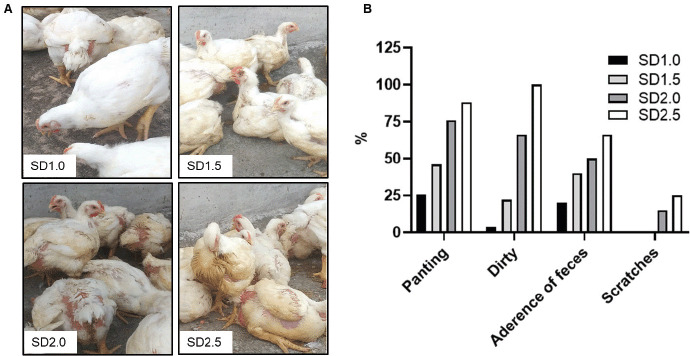
Effects of different SD rates on welfare. Broiler birds were reared in different SD spaces and welfare indicators were observed from 10 to 31 days. A. Representative pictures of broilers reared in different SD spaces at day 28. Birds reared in SD1.0 showed a clean and bright appearance. SD2.0 and SD2.5 birds have dirty appearances, feces adherences below the vent, and panting behavior. B. The graph shows the percent of panting, dirty, adherence of feces, and scratches in broilers reared in different SDs.

The evaluation of broiler welfare indicators was done by looking at a variety of parameters like litter status, the degree of bad smell, and dirtiness of body sides, wing, back, breast, feathers, leg lesions, hematomas, foot-pad dermatitis, scratches, and breast blisters in live birds and mortality rate, etc. The observations are recorded from 11 days of age and are presented in Table 1 and Figure 1. During the first 2 weeks, these indicators and the feeding and drinking behaviors were not significantly different among the birds of varying SD groups. As the birds’ ages progressed from 3 weeks, the birds in the SD2.0 and SD2.5 groups showed increased panting breathing during the day (11 a.m.–4 p.m.), with wet feces adhering below the vent of the majority of the birds. A significant number of birds had noticeably dirty bodies and soiled feathers (Table 1 and Fig. 2). Foot-pad dermatitis, scratches, and blister formation in the leg were also observed in a few birds, particularly in group D. Litters of groups C and D frequently got moist within a week of replacing dry fresh litters (Fig. 2). Ammonia concentrations were observed by bad-smelling. The visitors were bound to block their nostrils. The present study did not detect any lameness, ascites, hematomas, or mortality among the birds of SD groups, although birds of groups C and D looked fatigued. It revealed that the SD negatively correlated to the welfare behavior indicators. The current findings are consistent with the findings of another study [20], which reported a reduction in broiler welfare at SD rates greater than 35 kg/m^2^. Other studies have found that higher SD is closely linked with less carousing, moving, preening, body care behavior, and feeding behavior [4]. High SD in challenged birds increased the gross lesion score in the intestine, contrary to unchallenged birds. High SD also harmed broiler chicks’ welfare and gut health in a subclinical trial as well as predisposed them to necrotic enteritis [21]. Foot-pad lesion and mortality rates are consistent with the previous findings [22]. Broiler chickens reared in 34 kg/m^2^ SD spaces have the highest mortality, the poorest walking ability, and the highest hock burn and pododermatitis [12].

**Table 1.** Effects of different SD rates on welfare indicators in broiler chickens.

Parameters	SD1.0		SD1.5		SD2.0		SD2.5	
Days	11–21	22–31	11–21	22–31	11–21	22–31	11–21	22–31
Status of litter	Dry	Dry and moist	Dry	Dry and moist	Moist	Moist	moist	Moist
The intensity of the bad smell	Low	moderate	Low	moderate	High	High	High	High
Panting (%)	0	26	14	45.5	51	76	54	80
Adherence of wet feces below vent (%)	0	20	0	40	0	50	54	66
Dirty (%)	0	4	18	22	59	66	54	100
Scratches and breast blisters (%)	0	0	0	0	0	15	0	25
Alertness (%)	100	100	100	100	100	100	100	100

### Effects of SD rates on growth performances

**Figure 3.**
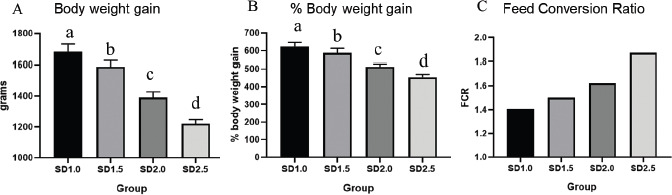
The effects of SD rates on (A) body weight gain (gm), (B) percent weight gain, and (C) FCR in broiler chickens on the 31st day of life. Values with dissimilar letters above the bar graph differ significantly (*p* < 0.05).

Table 2 and Figure 3 show the effects of different SD rates on live body weight, weight gain, and FCR. The initial body weight of broiler chicks (10th day of age) before separation into different SD spaces varied slightly but was statistically non-significant (*p* > 0.05). After 7 days, the live body weight of groups A and B differed significantly from the other groups (*p* < 0.05). On the 24th day, the lowest average live body weight was recorded in group D, following a similar pattern to the 17th day of age. There was a sharp rise in the body weight of the lower density group (SD1.0) from days 17 to 31 of the experiment. The highest average body weight was recorded in birds reared in the lower SD group, and the lowest was recorded in the birds of the higher SD group. Among the groups, all data were statistically significant (*p* < 0.05). The results clearly show that SD is an important factor that directly affects the weight of the broiler birds.

**Table 2.** Effects of different SD rates on live body weight (gm) (Mean ± SEM) in broiler chickens.

Group	Live body weight (gm) (Mean ± SEM)			
	10th day	17th day	24th day	31st day
SD1.0	271.78 ± 4.55	681.42 ± 17.21^a^	1,251.42 ± 31.57^a^	1,951.78 ± 50.01^a^
SD1.5	275.25 ± 6.10	671.13 ± 12.11^a^	1,230.68 ± 15.50^a^	1,832.64 ± 34.51^b^
SD2.0	271.20 ± 4.75	569.65 ± 13.94^b^	1,063.10 ± 33.11^b^	1,662.96 ± 35.47^c^
SD2.5	274.86 ± 6.47	564.86 ± 11.16^b^	1,025.97 ± 16.84^b^	1,496.52 ± 24.86^d^

^a,b,c,d^ Values with superscript letters in a column differ significantly (*p* < 0.05).

The body weight gain and body weight gain % (Fig. 3) of each group were calculated as per the formula, and the maximum body weight gain (mean value) was noticed in group A and the lowest in group D. In terms of FCR, birds in group A (SD1.0) had an FCR of 1.40, group B (SD1.5) had an FCR of 1.50, group C (SD2.0) had an FCR of 1.62, and group D (SD2.5) had an FCR of 1.87. Our investigation revealed that broilers reared at a lower SD rate have a lower effect on FCR than birds reared at higher SD. When broiler chickens are allocated more space, feed and water intake are appropriately utilized. On the other hand, feed and water intakes were negatively affected by the increased rate of SDs. Notably, higher SD was associated with lower feed consumption. This could be due to increased competition for the feeding space. Interestingly, high SD has previously been shown to significantly decrease broiler growth rate and increase wet litter and foot-pad and thigh lesion incidences [6,13]. The present findings support these observations. The live body weight, weight gain, and FCR of broilers all improved as the SD rate decreased during the period of the experiment. Accessibility to feeders and drinkers was most likely restricted due to increased SD as well as competition between birds for access to the feeder. Birds reared at an SD of 5 birds/m^2^ had better growth than those reared at 10 birds/m^2^. The birds raised in SD at 15 birds/m^2^ gained the least weight [23]. High SD (12 birds/m^2^) decreased the body weight and growth performance of growers and (or) finishers of male Cornish Cross cockerels [24]. Furthermore, a SD of 10 chicks per square meter resulted in the highest production index [25]. Compared to low SD-raised chickens, high SD-raised chickens showed a decrease in body weight gain and feed intake in the starter and whole phases and increased FCR in the finisher phase [26]. The current findings are similar to those of Yu et al. [27] and Goo et al. [28], who found that broilers reared at high SD spaces (18 birds/m^2^) had lower live body weight, body weight gain, and feed consumption, as well as impaired intestinal barrier function, without affecting meat quality or anti-oxidant conditions in the liver. In terms of muscle quality, the high SD group had significantly lighter breast muscles 45 min and 24 h after slaughter [29]. The capacity of the breast muscle to hold water was reduced, but the springiness of the breast and thigh, as well as the hardness of the thigh, were increased [24].

**Table 3.** Effects of different SD rates on biochemical parameters in broiler chickens.

Group	SD1.0	SD1.5	SD2.0	SD2.5
TC (mg/dl)	146.25 ± 3.81	144.04 ± 4.36	138.09 ± 3.73	147.60 ± 8.67
TG(mg/dl)	92.31 ± 2.21	83.47 ± 1.44	89.89 ± 2.99	91.85 ± 2.24
LDL-c(mg/dl)	100.31 ± 1.22	101.6 ± 2.56	102.51 ± 2.60	115.89 ± 1.88
HDL-c(mg/dl)	43.07 ± 1.17	42.17 ± 1.67	40.01 ± 1.52	39.94 ± 0.87
AST (U/l)	10.95 ± 0.74	11.02 ± 0.52	11.21 ± 0.48	11.076 ± 0.48
ALT(U/l)	4.14 ± 0.23	4.37 ± 0.66	4.35 ± 0.10	4.75 ± 0.12
Creatinine (mg/dl)	0.98 ± 0.04	1.03 ± 0.07	1.06 ± 0.06	1.03 ± 0.05
Total protein (gm/dl)	4.88 ± 0.31^a^	3.96 ± 0.18^b^	3.57 ± 0.21^b^	3.19 ± 0.17^b^

^a,b^ Values with different superscript letters in a column differ significantly (*p* < 0.05).

**Figure 4.**
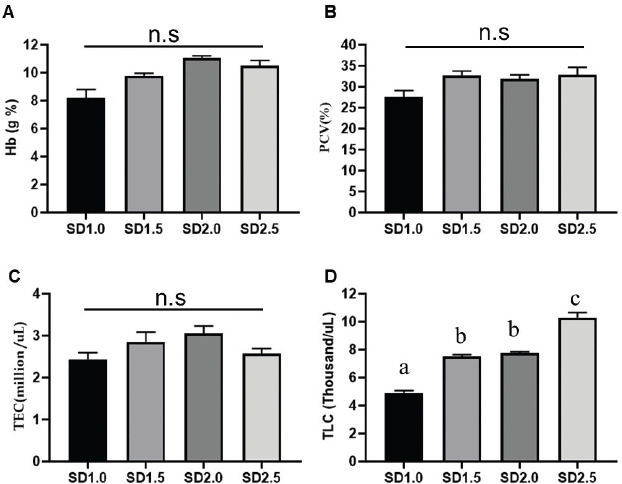
Effects of different SD rates on (A) Hb concentration (gm%), (B) PCV (%), (C) TEC (million/μl) and (D) TLC (thousand/μl) in broiler chickens at 31st day of age. *Values with dissimilar letters above the bar graph differ significantly (*p* < 0.05), n.s- not-significant.

### Effects of SD on hemato-biochemical profile

Results showed that the Hb concentration in group A (SD1.0) was 9.21 ± 0.59 gm%, in group B (SD1.5) was 9.8 ± 0.16 gm%, in group C (SD2.0), it was 11.06 ± 0.17 gm% and in group D (SD2.5) was 10.50 ± 0.38 gm% (Fig. 4). Although the values were slightly higher in groups C and D, the data were statistically insignificant (*p* > 0.05). The mean values of TEC for group A were 2.93 ± 0.16 million/μl, in group B, it was 2.84 ± 0.24 million/μl, in group C, it was 3.05 ± 0.18 million/μl, and in group D, it was 2.57 ± 0.12 million/μl respectively. The data were not statistically significant (*p *> 0.05). The mean values of PCV in group A were 30.52% ± 1.55%, in group B was 32.7% ± 1.03%, in group C, it was 31.85% ± 0.99%, and in group D, it was 32.93% ± 1.70%. The data were not statistically significant among the birds. The values of hematological parameters remained within the normal range. It showed that different SD rates did not alter the hematology in broilers in our current experimental methodology. On the other hand, the total leucocyte count increased significantly (*p* < 0.05) in the birds reared in higher SD (Fig. 4). 

The lipid profile, including TC, TG, HDL-c, and low density lipoprotein–cholesterol (LDL-c) levels in the blood sera of the birds reared at different SD rates, were found to be similar and statistically non-significant (Table 3). The mean ALT and AST levels are almost similar and statistically insignificant among the groups. The mean creatinine levels of different groups were also found to be more or less comparable and not statistically significant (*p* < 0.05). However, the mean serum total proteins gradually decreased with the increase in the SD rates (Table 3). The values were statistically significant (*p* < 0.05) among the groups. Overall, the current findings using the current methodology show that, with the exception of TLC and total protein, birds reared in different SDs have little to no influence on their hemato-biochemical profile. When animals or birds are subjected to stress or disease conditions, their leukocyte counts rise. Birds reared in higher stocking densities are facing stress. The results are partially consistent with the previous reports stating that SD did not affect total serum protein and glucose concentration. Plasma corticosterone, serum glucose, cholesterol, total nitrites, and the heterophil/lymphocyte ratio were unaffected [6]. Another study found that broilers raised on higher SD had higher blood heterophil lymphocyte ratios (H: L) and corticosterone levels [29,27]. The PCV values were reduced, and serum AST concentrations were increased significantly (*p* < 0.05) by the increased rate of SD [17].

On the contrary, the current findings differed from the previous findings [25], indicating that SD had significant (*p* < 0.05) effects on serum glucose, uric acid, total protein, TC, TG, HDL-c, LDL-c, AST, and ALT levels. It was reported that semi-arid climates with 15 chicks/m^2^ SDs and mild and humid climates with 20 chicks/m^2^ SD treatments had the highest cholesterol and TG levels. On the other hand, the semi-arid climate with 17 chick/m^2^ SD treatment produced the highest HDL, the semi-arid climate with 20 chick/m^2^ SD treatment had the highest AST, and the hot and dry climate with 20 chick density treatment had the highest ALT (*p* < 0.05). The SD influenced the serum biochemical parameters [26].

## Conclusion

Consumers believe that SD is one of the most significant factors influencing animal welfare. The World Organization for Animal Health (OIE) prioritizes animal welfare. There is also a financial incentive to ensure animal welfare. The findings of the present study demonstrated that the welfare indicators were compromised in the broilers reared in moderate density (SD2.0) and high-density groups (SD2.5), which further hindered the growth performance as body weight, body weight gain, feed intake, and total serum protein were significantly (*p < *0.05) decreased in higher SD rates. Broilers housed in one (SD1.0) and one and a half (SD1.5) birds per square foot area performed better physiologically, ensuring animal welfare practice.

## List of abbreviations

ALT, Alanine aminotransferase; AST, Aspartate aminotransferase; FCR, Feed conversion ratio; Hb, Hemoglobin; HDL-c, High density lipoprotein-cholesterol; LDL–c, Low density lipoprotein–cholesterol; PCV, Packed cell volume; SD, Stocking density; TC, Total cholesterol; TEC, Total erythrocyte count; TLC, Total leukocyte count.
